# Coding of relative size in monkey inferotemporal cortex

**DOI:** 10.1152/jn.00907.2014

**Published:** 2015-01-14

**Authors:** T. Vighneshvel, Sripati P. Arun

**Affiliations:** Centre for Neuroscience, Indian Institute of Science, Bangalore, India

**Keywords:** neural coding, object recognition, perceptual constancy

## Abstract

We seldom mistake a closer object as being larger, even though its retinal image is bigger. One underlying mechanism could be to calculate the size of the retinal image relative to that of another nearby object. Here we set out to investigate whether single neurons in the monkey inferotemporal cortex (IT) are sensitive to the relative size of parts in a display. Each neuron was tested on shapes containing two parts that could be conjoined or spatially separated. Each shape was presented in four versions created by combining the two parts at each of two possible sizes. In this design, neurons sensitive to the absolute size of parts would show the greatest response modulation when both parts are scaled up, whereas neurons encoding relative size would show similar responses. Our main findings are that *1*) IT neurons responded similarly to all four versions of a shape, but tuning tended to be more consistent between versions with proportionately scaled parts; *2*) in a subpopulation of cells, we observed interactions that resulted in similar responses to proportionately scaled parts; *3*) these interactions developed together with sensitivity to absolute size for objects with conjoined parts but developed slightly later for objects with spatially separate parts. Taken together, our results demonstrate for the first time that there is a subpopulation of neurons in IT that encodes the relative size of parts in a display, forming a potential neural substrate for size constancy.

because the very act of image formation destroys depth, many accounts of size constancy require additional knowledge about the external world. A simpler possibility, however, is that size constancy can be achieved by comparing the size of the retinal image with that of other objects (Palmer 1999; Rock and Ebenholtz 1959). Encoding of relative size may also be useful in object vision in general, because it might serve to distinguish between highly similar objects.

To investigate this issue, we targeted the monkey inferotemporal cortex (IT), an area critical for object recognition, where neurons are tolerant to changes in size, position, and clutter (DiCarlo et al. 2012; Tanaka 1996). In previous studies, neurons were tested on a diverse set of shapes and were found to be selective for the same shape across changes in retinal size (Hikosaka 1999; Ito et al. 1995; Lueschow et al. 1994). This size tolerance could have been observed if neurons were responding to a specific feature without regard for the size of other features in an object or if neurons were responding to the relative size of two features in an object. Thus previous reports of size tolerance in IT do not distinguish between these possibilities.

To assess whether IT neurons encode relative size, we devised highly simplified displays containing two parts—one large and one small—whose sizes were varied independently. Each part was presented at two possible sizes (1*x* or 2*x*), resulting in a total of four versions per object (see Fig. 2). Throughout, we denote these versions as v11, v12, v21, and v22 (where, e.g., v12 represents the version with the small part at size 1*x* and the large part at size 2*x*). In this design, a neuron sensitive to the size of a single part would change its response whenever that part's size was changed regardless of the size of the other part. For instance, the response difference between v11 and v12 would be the same as the difference between v21 and v22, because in both cases the larger part increased in size. This would produce a main effect in an analysis of variance (ANOVA) on the response with the sizes of the two parts as factors. In contrast, a neuron sensitive to relative size of the two parts would respond similarly when parts are scaled in proportion. Thus it would respond more similarly to v11 and v22—which represent both parts scaling 2*x* in size—than to other versions such as v12 and v21 where the parts scale disproportionately. This would produce an interaction effect in the same ANOVA. Although nonlinear interactions between parts of an object have been reported previously in IT neurons (Brincat and Connor 2006; Miller et al. 1993; Sripati and Olson 2010), it is not known whether there are nonlinear interactions between the size of parts in a display. Our results show that while the majority of IT neurons are modulated by the absolute size of parts, a subpopulation of neurons encodes the relative size of parts in a display, forming a potential neural substrate for size constancy.

## MATERIALS AND METHODS

### 

#### Neurophysiology.

All procedures were performed according to an experimental protocol approved by the Institutional Animal Ethics Committee of the Indian Institute of Science, Bangalore and the Committee for the Purpose of Control and Supervision of Experiments of Animals, Government of India. Single-unit activity was recorded from the IT of two adult male monkeys (*Macaca radiata*; laboratory designations *Ro* and *Ka*, ages 12 and 8 yr). Each animal was surgically implanted with a titanium headpost and subsequently with a recording chamber positioned vertically above IT. The location of the recording chamber was determined with structural MRI and was centered over the anterior half of left IT. It was subsequently verified through the phasic transitions and anatomical landmarks during recording in both monkeys and with structural MRI after surgery in *monkey Ka*. The recording locations corresponded to the ventral bank of the superior temporal sulcus and the inferior temporal gyrus lateral to the anterior middle temporal sulcus (AMTS) (centered at levels A14, L19 mm in *Ro* and A12, L17 mm in *Ka* relative to the interaural plane and midline). On each day of recording for *monkey Ro*, a tungsten microelectrode (FHC) was lowered into the brain with a micromanipulator (Narishige) with the aid of a stainless steel guide tube. For *monkey Ka*, we used a 24-channel U probe (100-μm contact spacing, Plexon). The electrode was then advanced until phasic visual responses were observed. Action potentials were recorded with a commercial amplifier (Omniplex system, Plexon) and were initially isolated with a high-pass filter (4-pole Butterworth filter, cutoff frequency 250 Hz). Subsequently they were sorted off-line into putative individual units with a spike-sorting software based on waveform and cluster analysis (Offline Sorter, Plexon). We recorded from a total of 103 visually responsive neurons (53 from *monkey Ro*, 50 from *monkey Ka*) and obtained qualitatively similar results using the data from each monkey separately.

#### Behavioral task.

All aspects of behavior were under control of a computer running Cortex software (NIMH DOS Cortex). Eye position was monitored with an infrared eye tracker system (ETL-250, ISCAN). Stimuli were presented on an LCD monitor (ViewSonic; 120 Hz refresh rate). Monkeys were trained to passively fixate a series of images presented at the fovea and received a juice reward for successfully maintaining fixation within a 3° window centered on a fixation cross. Post hoc analyses revealed that the monkeys' gaze was closely centered around fixation (average standard deviations in the *x* and *y* directions: 0.21° and 0.34°), with no systematic difference across versions. Each trial began with a fixation cross that was replaced by six rapidly flashed stimuli (200 ms on and off) presented in random order (see below). Error trials were repeated after a random number of other trials.

#### Stimuli.

**Fig. 1.**
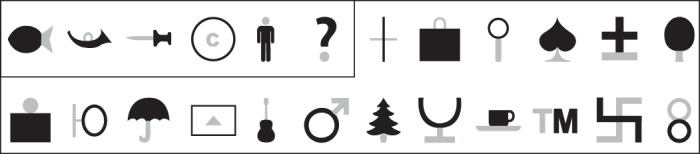
Shapes used to probe neuronal encoding of relative size. Each neuron was tested on a total of 12 stimuli, which always included the first 6 (enclosed by a box), with the remaining 6 chosen to evoke a broad range of responses. All shapes are shown in their version v11/v22. The small part is shown in gray for visualization here, but in the actual experiment stimuli were fully white against a black background.

**Fig. 2.**
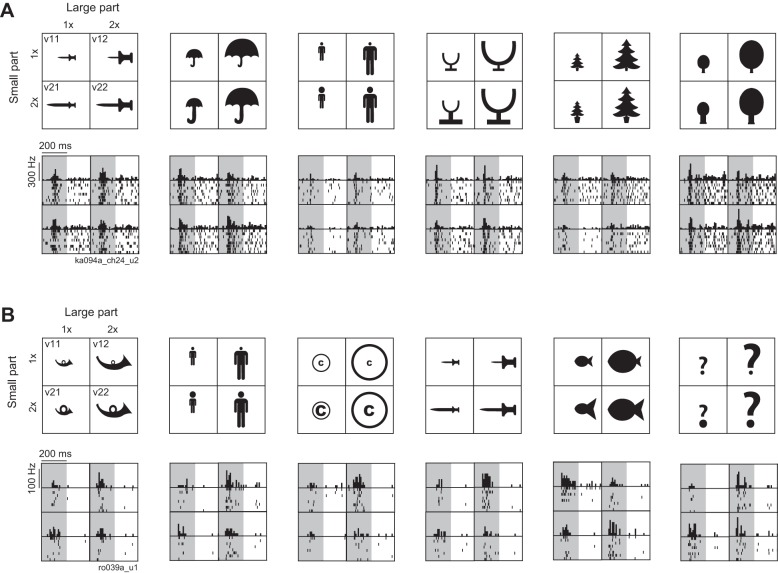
Example responses of 2 inferotemporal cortex (IT) neurons (*A* and *B*) to a subset of the stimuli. *Top*: each shape consisted of 2 parts that were shown at 2 sizes (1*x* or 2*x*), resulting in a total of 4 versions (labeled v11, v12, v21, v22 for shapes on *left*). Responses are shown at *bottom*, with black bars representing the firing rate across time and ticks indicating spike times (1 row per trial). The gray region is the image presentation period (200 ms).

A total of 12 objects were used for each neuron—6 of them were common to all neurons (Fig. 1), and the remaining 6 were chosen for each neuron out of a library of 18 objects to evoke a broad range of responses (for version v22). Each object was scaled to have a longer dimension of 3° in its smallest version (v11) and contained two parts that could be either conjoined or separate, with one part typically larger than the other (median ratio of large to small part area = 2.8). Version v22 was centered on fixation, and the junction/space between the parts was kept at a fixed retinal location while the two parts on either side were scaled in size (as shown in Fig. 2). In all, there were 19 conjoined objects and 5 objects with spatially separate parts. Post hoc analyses revealed no qualitative difference in the frequency of interactions between these two groups.

#### Trial design.

For 24 of the recorded neurons, the six stimuli in a trial were chosen such that no two stimuli were derived from the same object, in order to avoid response adaptation. However, in this design there were drastic changes in image size in a trial. To avoid such large changes, we imposed a second constraint that stimuli in a given trial should all be derived from the same version across objects. The remaining neurons in the experiment were recorded in this manner. Post hoc analyses revealed qualitatively similar results for both groups of neurons.

#### Multidimensional scaling analysis.

To visualize similarity relations between the stimuli, we first calculated a measure of population dissimilarity for each pair of versions, which was simply the absolute difference in firing rate elicited by the two versions averaged across the relevant population, in a window of 70–270 ms after image onset (other time intervals yielded similar results). This measure represents the extent to which the two versions elicited similar patterns of activity across neurons. This results in a total of ^4^C_2_ = 6 distances for each object. We then averaged these distances across all instances of main or interaction effects to obtain an average measure of distance between versions. To visualize these distances, we performed multidimensional scaling, which finds a set of two-dimensional (2D) coordinates for each version such that the 2D distances are as close as possible to the observed distances. This was implemented with built-in functions in MATLAB (*mdscale*).

#### Assessment of visually driven activity for each shape across versions.

In the ANOVA performed for each shape across versions with the size of each part as factors, the lack of any significant effect could have arisen because of a zero response to all versions (because that shape is not preferred by the neuron) or may have evoked a constant nonzero response. The latter possibility is interesting because it indicates that the neuron may be invariant to changes in size of either part regardless of their relative proportions. To assess this possibility, for each case, we compared the firing rate across all four stimuli in the spontaneous period (from 50 ms before to 50 ms after image onset) and in the image-on period (from 50 to 150 ms after image onset). This comparison showed that 61% (530 of 869) of the cases with no significant effect in the ANOVA had a significant visual response (Wilcoxon signed-rank test, criterion = 0.05).

#### Unbiased estimates of ANOVA effect strengths.

For each neuron and object, we performed an ANOVA on the firing rates during the stimulus presentation time (bin size of 20 ms) with the sizes of the large part (1*x* or 2*x*) and small part (1*x* or 2*x*) as factors. A direct measure of main effect strength would be the difference between responses to preferred and nonpreferred sizes. However, this measure is biased because it will always yield a positive difference, even for random noise. To overcome this limitation, we used a split-half approach. Using the odd-numbered trials we identified the preferred size and then used the even-numbered trials to calculate the difference between the responses to the preferred and nonpreferred sizes. This process was repeated by identifying the preferred part size using the even-numbered trials and calculating the response difference using the odd-numbered trials. These two estimates were averaged together to yield an unbiased estimate of the effect strength. This method ensures that the average effect strength is zero for random noise. We used the same approach to calculate unbiased estimates of interaction effect strengths.

## RESULTS

We recorded from 103 visually responsive neurons from the left IT cortex of two monkeys (53 from *monkey Ro*, 50 from *monkey Ka*) while they performed a fixation task. Each neuron was tested on a total of 48 stimuli comprised of 12 shapes (selected from a set of 24; Fig. 1) presented in 4 versions each. Each shape consisted of a large part and a small part that could be conjoined or spatially separated. Each part could have two possible sizes (1*x* or 2*x*), resulting in four versions for each shape (Fig. 2). Throughout the text, these versions are denoted as v11, v12, v21, and v22 (e.g., v12 represents the stimulus with the small part at size 1*x* and the large part at size 2*x*).

To investigate whether neurons encode the relative size of parts, we performed an ANOVA on the firing rate evoked by the four versions of each shape (during the 200-ms image presentation period), with the sizes of the large (1*x*/2*x*) and small (1*x*/2*x*) part as factors. As described earlier, main effects represent sensitivity to absolute size of a part, whereas interaction effects represent nonlinear interactions between the two part sizes. Of particular interest to us was whether these interactions resulted in similar responses to the two proportionately scaled versions (v11 and v22), which reflect encoding of relative size.

There was considerable diversity in the response patterns both within single neurons (across shapes) and across neurons. Figure 2 illustrates the responses of two IT neurons that highlight this diversity. The first neuron was mostly sensitive to the absolute size of the parts but showed a variety of response patterns across shapes (Fig. 2*A*). For the first and second shapes from the left in Fig. 2*A*, it showed no significant response modulation across the four versions (*P* > 0.05 for main and interaction effects). For the third and fourth shapes in Fig. 2*A*, this neuron exhibited a main effect of the large part (*P* < 0.05). For the fifth shape in Fig. 2*A*, its responses were independently modulated by the size of both parts (*P* < 0.005 for both main effects) but there was no significant interaction. Note that although such a combination of main effects can produce similar responses to the proportionately scaled versions, such instances were extremely rare across neurons and shapes (*n* = 10 instances across 103 × 12 = 1,236 tetrads). For the sixth shape in Fig. 2*A*, this neuron showed a main effect of the large part and an interaction effect (*P* < 0.05), resulting in similar responses to the two proportional versions (v11 and v22). Overall, however, this neuron exhibited similar tuning to shapes across versions: in other words, if it responded strongly to a particular shape in, for example, version v11, it was also likely to do so for all other versions. To quantify this observation, we calculated the correlation in the firing rate across all 12 shapes tested for every pair of versions—and found a very high correlation for all version pairs (*r* = 0.93, 0.92, 0.95, 0.88, 0.93, 0.88 for v11–v12, v11–v21, v11–v22, v12–v21, v12–v22, v21–v22, respectively; all correlations significant at *P* < 0.0005).

The second neuron showed several instances of sensitivity to relative size (Fig. 2*B*). It showed no significant response modulation to the first shape in Fig. 2*B* but showed a significant main effect for the large part for the second shape (*P* < 0.05). For the third shape in Fig. 2*B*, it only responded to one of the versions (v12), indicative of two main effects and an interaction effect. Such a combination of main and interaction effects could in principle cause similar responses to proportionately scaled versions, provided the single strong response occurs for v12 or v21. However, this pattern was too infrequent across neurons and shapes to permit detailed testing (*n* = 4 instances across 1,236 tetrads tested). This neuron also produced similar responses to proportionately scaled versions for the fourth and fifth shapes in Fig. 2*B*. In contrast, for the sixth shape in Fig. 2*B* it showed the opposite pattern: a pure interaction effect (*P* < 0.05), with similar responses for the disproportionate versions (v12 and v21). Thus this neuron exhibited inconsistent shape tuning across versions—indeed, none of the pairwise tuning correlations was significant across the 12 shapes tested (*r* = −0.44, 0.1, 0.23, −0.51, 0.29, 0.19; *P* > 0.05 for all 6 version pairs).

Considering the diversity in the neuronal response to shapes across versions, we performed two types of analyses. First, we asked whether shape selectivity of each neuron was preserved across versions. Second, we treated each set of responses to the four versions of a shape as independent samples and looked for interaction effects consistent with coding for relative size. These are detailed below.

### 

#### Do IT neurons maintain their shape selectivity across versions?

**Fig. 3.**
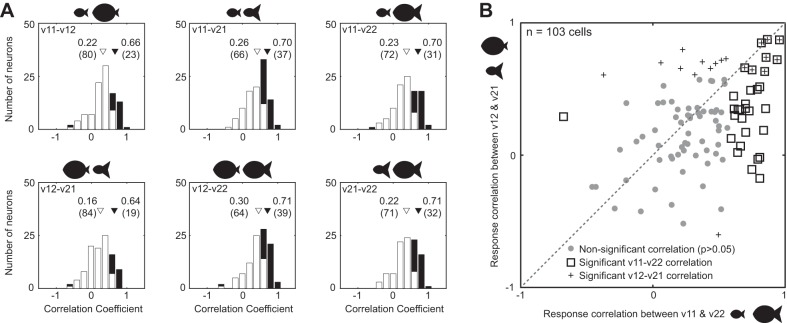
Consistency in shape selectivity across versions. *A*: distribution of correlation coefficients between the responses to the 12 shapes for every possible pair of versions. White bars represent statistically insignificant correlations, and black bars represent significant correlations (*P* < 0.05). *B*: response correlation between versions v11 and v22 plotted against response correlation between versions v12 and v21, across all 103 neurons.

We started by asking whether neurons tended to maintain their shape selectivity across versions. We calculated the correlation between the responses to the 12 shapes between every pair of versions, as described above for the example neurons. The distribution of correlations for every pair of versions is shown in Fig. 3*A*. The average correlation between every pair of versions was positive (*P* < 0.00005, Wilcoxon rank sum test), and the average significant correlation was very high for all pairs (*r* = 0.66, 0.71, 0.70, 0.65, 0.71, 0.71 for versions v11-v12, v11-v21, v11-v22, v12-v21, v12-v22, v21-v22, respectively, at a criterion of *P* < 0.05). Thus IT neurons maintain their shape selectivity overall despite distortions introduced by variations in part size.

#### Do IT neurons show similar tuning to proportionately scaled parts?

Although IT neurons maintained their shape selectivity across versions, we wondered whether they tended to respond more similarly to proportionately scaled versions than to disproportionately scaled versions. Of particular interest to us was whether neurons responded more similarly to versions v11 and v22 (the proportionate versions) compared with versions v12 and v21 (the disproportionate versions). This is a very interesting comparison for the following reason: consider a neuron that is modulated by the absolute size of the parts. Suppose it changes its firing rate by *Δ_1_* when *part 1* doubles in size, and by *Δ_2_* when *part 2* doubles in size. Then the difference in the response between v11 and v22 will be *Δ_1_* + *Δ_2_*, whereas the difference in response between v12 and v21 will be *Δ_1_* − *Δ_2_*. Such a neuron will therefore produce a large change in response between v11 and v22 and a small change in response between v12 and v21. This in turn implies a stronger correlation between v12 and v21 than between v11 and v22, especially considering that part size modulations are diverse (Fig. 2). In contrast, a neuron that is modulated by the relative size of the two parts should show a stronger correlation between v11 and v22 than between v12 and v21. To assess this possibility, we plotted the response correlation between v11 and v22 against that of v12 and v21 (Fig. 3*B*). Across the population of 103 cells, 31 cells showed a significant response correlation (*P* < 0.05) for versions v11 and v22, but only 19 did so for versions v12 and v21. Across cells with consistent tuning for either v11-v22 or v12-v21 (*n* = 42), v11-v22 response correlations were significantly larger than for v12-v21 (average correlations: 0.60 for v11-v22, 0.42 for v12-v21; *P* = 0.003, Wilcoxon signed-rank test).

The above argument shows that a neuron encoding absolute size will produce the greatest response modulation between v11 and v22 and the least modulation between v12 and v21. It can also be extended to other comparisons such as v11-v22 versus v11-v12, but these response modulations may not differ by as much. For instance, the change in response will be *Δ_1_* + *Δ_2_* between v11 and v22 but only *Δ_1_* between v11 and v12. As a result, a difference in tuning correlation may be harder to detect. This is indeed what we found: across the same 42 cells as above, the v11-v22 correlations were larger in magnitude than other version pairs, but this difference was either statistically significant or approached it (average correlations: 0.43, 0.53, 0.60, 0.42, 0.53, 0.52 for pairs v11-v12, v11-v21, v11-v22, v12-v21, v12-v22, v21-v22; *P* values of a Wilcoxon signed-rank test between v11-v22 and the other 5 pairs v11-v12, v11-v21, v12-v21, v12-v22, v21-v22: 0.004, 0.06, 0.003, 0.17, 0.03, respectively). We conclude that IT neurons tend to respond more similarly to proportionately scaled versions than to disproportionate ones.

#### Do IT neurons show similar responses to proportionately scaled parts?

As detailed above, our stimuli were constructed such that absolute and relative size encoding would produce different response patterns. But can similar responses to versions v11 and v22 be explained by simple pixel-level differences? To confirm that this is not the case, for each pair of versions of a given shape we calculated the summed absolute difference between pixels of the two versions after scaling the net intensity of each image to 1. Versions v11 and v22 had the largest pixel difference compared with all other versions (average pixel difference across 24 objects: 0.83, 1.44, 1.56, 1.50, 1.17, 0.41 for v11-v12, v11-v21, v11-v22, v12-v21, v12-v22, v21-v22, respectively; *P* < 0.0005 in a Wilcoxon signed-rank test on a comparison between v11-v22 and all other pairs). Thus neurons that responded simply to the pixel-level differences in the image would elicit the greatest difference in firing rate for versions v11 and v22 compared with all other pairs of versions. In contrast, neurons encoding relative size would elicit the smallest difference in firing rate for precisely these versions.

To address these issues, we analyzed the responses to the four versions of each shape separately for each cell. This yielded a total of 1,236 response tetrads (103 neurons × 12 shapes). To investigate whether responses varied systematically across versions, we calculated the firing rate for each tetrad normalized by the maximum across versions to factor out variations due to shape. We observed a systematic overall modulation by absolute size: version v11 (both parts at size 1*x*) typically elicited the least firing rate, followed by v21 (small part at 2*x*), v12 (large part at size 2*x*), and v22 (both parts at 2*x*) (Average normalized rates: 0.73, 0.77, 0.83, 0.82 for versions v11, v21, v12, v22, respectively, across 1,236 tetrads; this variation was significant in a 1-way ANOVA, *P* < 0.0005, average firing rate: 14 spikes/s).

**Fig. 4.**
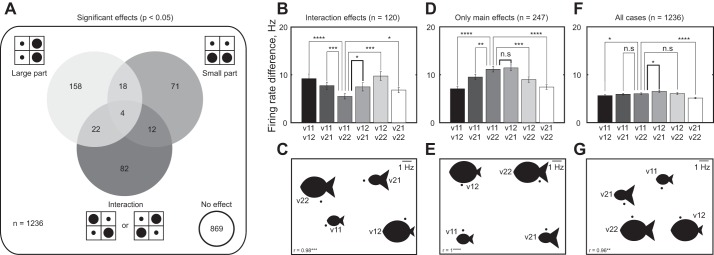
Encoding of relative size in IT neurons. *A*: distribution of significant effects in an ANOVA performed on each set of 4 versions (103 neurons × 12 objects = 1,236 cases) with large and small part size as factors. *B*: for interaction effects (*n* = 120), the average absolute difference in firing rate (70–270 ms) is plotted for each pair of versions. Version labels are given in the plot below. Statistical significance: *P < 0.05, **P < 0.005, etc. *C*: multidimensional scaling plot representing the same data as in *B*. In the plot, versions that evoked similar activity are placed nearby. The fish versions are shown for illustration, but the data are derived from all interactions across shapes. Values at *bottom left* of the plot indicate the correlation between the observed firing rate differences and the 2-dimensional (2D) plot coordinates. The high correlations show that the 2D plot is an accurate visualization of the actual firing rate differences. Note that all statistical analyses in *B* are based on the observed firing rate differences and this plot is only for visualization purposes. *D* and *E*: plots similar to *B* and *C* but for cases with only main and no interaction effects (*n* = 247). *F* and *G*: plots similar to *B* and *C* but for all the cases (*n* = 1,236).

To investigate these response patterns for each tetrad, we performed an ANOVA on the firing rate with the sizes of the large part (1*x*/2*x*) and the small part (1*x*/2*x*) as factors. The distribution of significant effects (criterion = 0.05) across all 1,236 cases is shown in Fig. 4*A*. We observed significant modulation due to part size in only 30% (367 of 1,236) of the cases. Of the remaining cases with no significant effect, ∼61% exhibited a statistically significant visual response to all versions (see materials and methods). Of the cases with a significant modulation by part size, 78% (285 of 367) exhibited main effects and 33% (120 of 367) exhibited a significant interaction effect. These interactions occurred in 55% (57 of 103 cells) of the recorded population. The incidence of interactions was similar in both monkeys (57 of 600 cases in *monkey Ka* and 63 of 636 cases in *monkey Ro* had interactions).

Are these interaction effects localized to a particular subpopulation of neurons or are they distributed randomly across neurons and shapes? To assess this possibility, we performed a split-half analysis: we separated the objects tested for each neuron into two groups and asked whether the presence of an interaction effect in one group of objects predicted the presence of an interaction in the other group. A negative result would imply that interactions are distributed randomly throughout neurons, whereas an affirmative answer would imply a tendency for interactions to co-occur within a neuron, implying the existence of a subpopulation of cells. To this end, we calculated the average statistical significance (i.e., average *P* value of the interaction effect in the ANOVA) for each object group for every neuron and asked whether the average interaction effect significance was correlated across the two groups of objects—this was indeed the case (*r* = 0.36, *P* < 0.0005). To confirm that this result is truly because of a tendency for interactions to co-occur, we performed a shuffle control in which stimuli and cells were randomly assigned and repeated the same analysis. This yielded no significant correlation (*r* = −0.09, *P* = 0.39). Thus interaction effects tended to co-occur within a subpopulation of neurons.

If IT neurons encode relative size, these interaction effects should cause proportionately scaled parts to elicit similar responses. To investigate this possibility, we calculated the absolute difference in the firing rate elicited by each pair of versions averaged across all interaction effects (in a window 70–270 ms after image onset). The two proportionately scaled versions (v11 and v22) elicited more similar activity compared with all other pairs (average absolute difference in firing rate between version pairs v11-v12, v11-v21, v11-v22, v12-v21, v12-v22, v21-v22: 9.2, 7.7, 5.5, 7.4, 9.8, 6.8 spikes/s, respectively; *P* < 0.05 on a Wilcoxon signed-rank test comparing v11-v22 with all other pairs; Fig. 4*B*). This effect was present in both monkeys and either attained or approached statistical significance (average absolute difference in firing rate for the 6 version pairs: 7.7, 6.8, 4.8, 6.7, 9.0, and 5.6 spikes/s for *monkey Ro*, *P* < 0.05 in a Wilcoxon signed-rank test comparing v11-v22 differences and all other version pairs except for v21-v22, where it was *P* = 0.16; firing rate differences were 10.5, 8.6, 6.1, 8.1, 10.4, 7.9 spikes/s for *monkey Ka*, *P* < 0.05 in a Wilcoxon signed-rank test comparing v11-v22 with all other pairs except with v12-v21, where it was *P* = 0.09).

To confirm that the statistical significance of these comparisons did not arise because of the disproportionate contribution of interactions from a few neurons, we repeated the statistical comparison by averaging the firing rate difference across the significant interactions within each neuron and then compared the firing rate differences between every pair of versions normalized to the maximum firing rate. Here too we found that the two proportionately scaled versions (v11 and v22) elicited the most similar activity compared with all other versions (normalized firing rate differences: 0.23, 0.23, 0.15, 0.21, 0.25, 0.21 for v11-v12, v11-v21, v11-v22, v12-v21, v12-v22, v21-v22; *P* < 0.05 for all comparisons with v11-v22, Wilcoxon signed-rank test).

To visualize these similarity relations, we performed a multidimensional scaling analysis on these pairwise differences. In the resulting plot, images are placed nearby if they elicited similar activity across neurons. The multidimensional scaling plot graphically shows the two proportionately scaled versions to be closest to each other compared with all other versions (Fig. 4*C*). We conclude that part-part interactions serve to make proportionately scaled displays similar.

To rule out the possibility that main effects encode relative size, we performed a similar analysis on cases with main but no interaction effects. Here, response differences were larger when both parts changed in size (Fig. 4*D*), and the underlying representation was strikingly different from the interaction effects (Fig. 4*E*). Thus the encoding of relative size cannot arise from sensitivity to changes in either part.

The above analyses shows that main effects reflect sensitivity to absolute size, whereas interactions reflect encoding of relative size. Which of these two effects dominates in the entire population? To answer this question, we performed the same analysis on all cases (*n* = 1,236). We found that the proportionate versions (v11 and v22) evoked more similar activity than the disproportionate versions (v12 and v21); they were not the most similar two versions as in the interaction effects (Fig. 4*F*). The multidimensional scaling plot (Fig. 4*G*) also reveals a similar pattern as with the main effects (Fig. 4*E*). Thus sensitivity to absolute size is predominant across the entire population.

#### Time course of relative size effects.

**Fig. 5.**
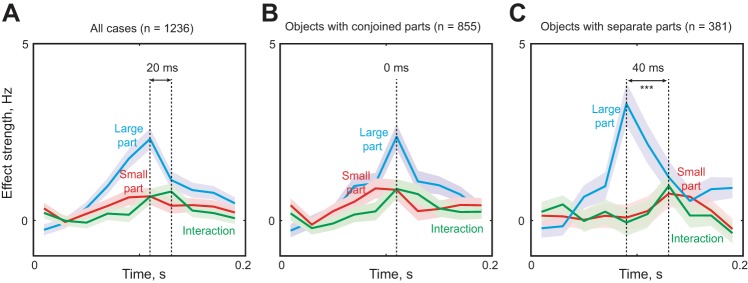
Time course of relative size encoding in IT. *A*: time course of main effect (cyan, large part; red, small part) and interaction effect (green) strengths averaged across all cases (*n* = 1,236 cases). Shaded regions represent SE. *B*: same plot as in *A* but for objects with conjoined parts (*n* = 855 cases). *C*: same plot as in *A* but for objects with spatially separate parts (*n* = 381 cases).

To investigate the dynamics of these interactions, we repeated the ANOVA analysis across the time course of the response and obtained unbiased estimates of the strengths of main and interaction effects (see materials and methods). We found that main effects attained a peak concomitant with the visual response (peak = 110 ms), whereas interaction effects peaked roughly 20 ms later (Fig. 5*A*). Could this difference in latency be due to the presence of two types of temporal patterns? We speculated that, since encoding of relative size involves interactions between the parts in a display, these interactions may take longer to develop if the parts are spatially separated.

To assess this possibility, we repeated the above analyses separately for objects with conjoined and spatially separated parts. There was no difference in the incidence of interactions in the two groups (10% of all 855 cases with conjoined parts; 9% of all 381 cases of spatially separated parts). However, the time courses of development of main and interaction effects in the two groups were different. For the cases with conjoined parts main and interaction effects attained a peak at the same time (*t* = 110 ms; Fig. 5*B*), whereas for spatially separate parts the interaction effects were delayed by 40 ms compared with the main effect of the large part but arose at the same time as the main effect of the small part (Fig. 5*C*). To assess the statistical significance of the latency difference, we performed a bootstrap analysis: we randomly sampled 50 neurons with replacement from the entire population of 103 neurons and calculated the peak latency for the main and interaction effects each time. The main effect latency for the large part was significantly smaller than the interaction effect latency (average latency: 90 ms for main, 130 ms for interactions; *P* < 0.0005, Wilcoxon signed-rank test across 50 bootstrap samples). This effect remained highly significant even when we compared the effect strength (averaged across stimuli) for each neuron. In contrast, the latency of the small part was not significantly different compared with the interaction effects (average latency: 130 ms for both; *P* > 0.05). We obtained qualitatively similar results upon varying the number of bootstrap samples and upon repeating these analyses for the data from each animal considered separately. We conclude that encoding of relative size is delayed when parts are spatially separate.

## DISCUSSION

The main finding of this study is that a subpopulation of neurons in monkey IT encodes the relative size of parts in a display. This encoding developed quickly for displays with conjoined parts and slightly later when the parts were spatially separate. Below we discuss these findings in the context of the known literature.

The finding that IT neurons encode relative size cannot have been anticipated from previous studies. This is because a size-invariant neuron as described by previous studies (Hikosaka 1999; Ito et al. 1995; Lueschow et al. 1994) could have been either sensitive to the absolute size of a part of the object tested or sensitive to the relative size of parts. It also cannot be explained by neurons tuned for junctions between parts (Sripati and Olson 2010), for two reasons: *1*) interactions were equally frequent when parts were conjoined or spatially separate and *2*) even if there were such a neuron, it would have to encode the relative size of contours on either side of the junction, which again amounts to encoding of relative size.

Here we have shown that encoding of relative size is a nonlinear interaction effect that cannot be explained by sensitivity to the absolute size of individual parts. While nonlinear interactions between parts of an object have been reported previously (Brincat and Connor 2006; Miller et al. 1993; Sripati and Olson 2010), to our knowledge this is the first report of nonlinear interactions between the sizes of two parts in a display. The fact that encoding of relative size develops later for spatially separate parts is concordant with the finding that nonlinear interactions between parts are generally weaker (Sripati and Olson 2010) and develop later (Brincat and Connor 2006). We speculate that interactions between object parts not only enhance selectivity (Brincat and Connor 2006; Sripati and Olson 2010) but also implement higher-order invariances such as relative size.

We have highlighted the fact that a subpopulation of IT neurons is sensitive to relative size, but this was not the predominant effect across the entire recorded population of neurons. The population tendency was toward strong positive correlation in shape selectivity across versions (Fig. 3) and sensitivity to the absolute size of parts (Fig. 4, *F* and *G*). However, our finding that a subpopulation of neurons is sensitive to relative size is likely to be a lower bound on its magnitude and incidence, considering that this was present in highly simplified displays in naive monkeys not required to make judgments of size. We speculate that encoding of relative size is far more prevalent during natural vision, particularly during judgments of real-world object size.

Our finding also raises intriguing questions regarding the underlying mechanisms. How can a neuron receiving size-dependent inputs become sensitive to relative size? If the inputs are size dependent, a proportional change in size of both parts should result in the largest change in response. Instead, this response is closer to that of the original object (Fig. 4, *B* and *C*). One possible mechanism could be that inputs arising from the two parts mutually inhibit each other. When the excitatory drive from one input increases, this leads to an overall increase in the response, but when excitation from both inputs increases, the net response reduces to the original level because the two inputs inhibit each other. This recurrent inhibition might be mediated by local inhibitory circuitry, which might explain the delayed onset of the interactions particularly for spatially separate parts (Fig. 5, *B* and *C*). To elucidate the mechanisms would require using individual parts presented in isolation as well as the two-part displays with interpart distance parameterized to reveal the influence of spatial separation on the encoding of relative size.

The fact that IT neurons encode relative size in highly simplified displays even in the absence of depth cues suggests that this may be a useful feature for object vision in general (Palmer 1999). It reflects the knowledge that objects that come closer scale proportionately rather than disproportionately. Encoding of relative size may also aid in making subtle distinctions within categories such as faces where objects may differ in the configuration of features rather than their identity. We propose that encoding of relative size may be an instance of a general principle whereby features that vary strongly with viewing conditions—such as brightness, position, and size—are encoded in relative terms in order to achieve perceptual constancy.

## GRANTS

This research was funded by an Intermediate Fellowship from the Wellcome Trust-DBT India Alliance, a startup grant from the Indian Institute of Science (IISc), and the DBT-IISc partnership programe (all to S. P. Arun).

## DISCLOSURES

No conflicts of interest, financial or otherwise, are declared by the author(s).

## AUTHOR CONTRIBUTIONS

Author contributions: T.V. and S.P.A. conception and design of research; T.V. and S.P.A. performed experiments; T.V. and S.P.A. analyzed data; T.V. and S.P.A. interpreted results of experiments; T.V. and S.P.A. prepared figures; T.V. and S.P.A. drafted manuscript; T.V. and S.P.A. edited and revised manuscript; T.V. and S.P.A. approved final version of manuscript.
